# Vegetation Productivity in Natural vs. Cultivated Systems along Water Availability Gradients in the Dry Subtropics

**DOI:** 10.1371/journal.pone.0168168

**Published:** 2016-12-22

**Authors:** Germán Baldi, Marcos Texeira, Francisco Murray, Esteban G. Jobbágy

**Affiliations:** 1 Grupo de Estudios Ambientales, IMASL, Universidad Nacional de San Luis & CONICET, San Luis, Argentina; 2 Laboratorio de Análisis Regional y Teledetección, IFEVA, Universidad de Buenos Aires & CONICET, Buenos Aires, Argentina; 3 Departamento de Métodos Cuantitativos y Sistemas de Información, Facultad de Agronomía, Universidad de Buenos Aires, Buenos Aires, Argentina; 4 A.E.R. San Luis, Instituto Nacional de Tecnología Agropecuaria, San Luis, Argentina; Estacion Experimental de Zonas Aridas, SPAIN

## Abstract

The dry subtropics are subject to a rapid expansion of crops and pastures over vast areas of natural woodlands and savannas. In this paper, we explored the effect of this transformation on vegetation productivity (magnitude, and seasonal and long-term variability) along aridity gradients which span from semiarid to subhumid conditions, considering exclusively those areas with summer rains (>66%). Vegetation productivity was characterized with the proxy metric “Enhanced Vegetation Index” (EVI) (2000 to 2012 period), on 6186 natural and cultivated sampling points on five continents, and combined with a global climatology database by means of additive models for quantile regressions. Globally and regionally, cultivation amplified the seasonal and inter-annual variability of EVI without affecting its magnitude. Natural and cultivated systems maintained a similar and continuous increase of EVI with increasing water availability, yet achieved through contrasting ways. In natural systems, the productivity peak and the growing season length displayed concurrent steady increases with water availability, while in cultivated systems the productivity peak increased from semiarid to dry-subhumid conditions, and stabilized thereafter giving place to an increase in the growing season length towards wetter conditions. Our results help to understand and predict the ecological impacts of deforestation on vegetation productivity, a key ecosystem process linked to a broad range of services.

## Introduction

Although the dry subtropics have been historically subject to a diverse array of human interventions, including logging, grazing, and cropping [1], in the last decades, a rapid expansion of agriculture took place over woodlands and savannas [2], leading to a deep environmental and human change [3]. This process was triggered fundamentally by the increasing overseas demand for food and fuel, technological improvements, the development of transport infrastructure in formerly remote areas, and the stabilization of local economies and politics [4].

Cultivation of crops and pastures often leads to shifts in ecosystem functioning through the modification of the composition and structure of vegetation and consequently, of resource acquisition strategies and potential growth rates [5,6]. In these regions, the few previous studies dealing with the biophysical consequences of this transformation showed small and non-significant differences in the magnitude of vegetation productivity, but strong contrasts in its seasonal and long-term variability [7,8].

In most of the classical and more recent empirical models of vegetation productivity drivers, climate interacts with vegetation structure dictating the temporal and spatial productivity patterns of natural ecosystems [6,9]. In particular, along water availability gradients–set by the balance between precipitation and evapotranspiration–, the average productivity shows a linear increase up to a threshold beyond which it levels off or decreases, likely because nutrient availability and/or solar radiation become the most limiting factors [5]. Also, strong effects on seasonality and inter-annual variability are described, particularly with the expansion of the growing season and the greater stability among years with increasing water availability [10–12].

Our understanding of the link between productivity and water availability has grown steadily in the last decades, focused initially on the water-use efficiencies [13] and later on the responses to global climate change [14]; however, much remains to be learned about vegetation productivity responses to land use transformations [15–17]. Notoriously, most studies have been biased towards North American and Asian temperate grasslands [12,18], with woody or agricultural systems being mostly overlooked [19,20]. These knowledge gaps are particularly critical as we try to integrate the effects of cultivation with those of climate on the multiple dimension of the Earth System functioning, among which vegetation productivity is one of the most critically connected with biogeochemical cycles and energy fluxes [5].

The aim of our study is to compare the vegetation productivity patterns of implanted crops and pastures (hereafter, cultivated systems) with the natural–predominantly woody–vegetation that they replace (hereafter, natural systems), across climatic water availability gradients in the dry subtropics (only those with summer rains). We focus our analysis on the magnitude, seasonality and inter-annual variability of vegetation productivity. Our guiding questions are: (1) How do key vegetation productivity attributes respond to cultivation?, and (2) How does this response vary along water availability gradients? The analyses are conducted at global and regional levels by means of the “Enhanced Vegetation Index” (EVI) and additive models for quantile regressions.

## Methods

### Study area

**Fig 1:**
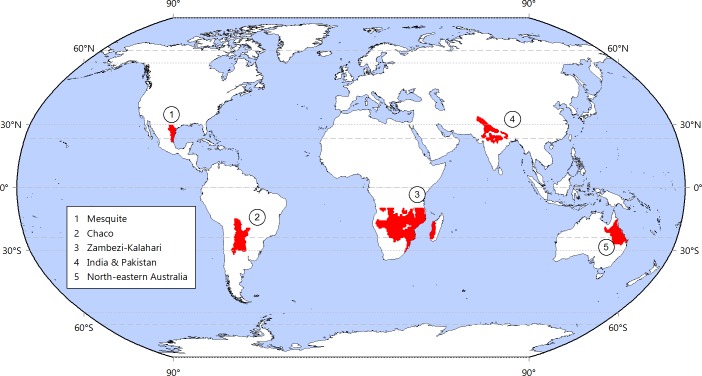
Study regions. Global distribution of dry subtropical systems with summer rains, defined by climatic and topographic features. Within these regions, we sampled natural and cultivated points along water availability gradients, encompassing semiarid to subhumid conditions.

We focused on the dry subtropics receiving summer rains, as defined by climatic and topographic features: warm temperatures (20 to 25°C of mean annual temperature), dry winters/wet summers (>66% of precipitation in the warm half of the year), semiarid to subhumid conditions defined by the ratio of mean annual precipitation to potential evapotranspiration (PPT:PET, from0.35 to 1.0), gentle slopes (<0.7%), and low elevation (<1200 m). Resulting regions were named as Chaco, India-Pakistan, Mesquite, North-eastern Australia, and Zambezi-Kalahari (Fig 1). These are predominantly uncultivated (except India & Pakistan) and show large differences in terms of population density, connectivity to markets, and affluence/technology [21].

### Sampling design

Across the study area, we generated a regular grid of sampling points distributed within 35 transects (20 km-wide and 125 to 250 km away from each other) that covered major PPT:PETgradients. These points, that maintained an approximate distance of 7 km with each other, were classified into two possible land use systems, natural or cultivated, based on a visual inspection of high resolution satellite images (“Google Earth”, http://www.google.com/earth/index.html) and online photographic archives (“Confluence Project”, http://www.confluence.org, and “Panoramio”, http://www.panoramio.com). Cultivated sampling points represented areas where natural vegetation was removed in order to implant artificial communities. We restricted all analyses to points with a homogeneous and constant land use/cover at the measurement scale and throughout the study period. We confirmed the homogeneity through the visual inspection of Google Earth images on 1.5 km-radius windows, while we resolved the constancy by restricting analyses to natural points characterized as such in 2012, and to cultivated points characterized as such in 2000 or earlier. These conditions were evaluated by visual inspection of circa 2000 imagery from the “GeoCover” Orthorectified Landsat ETM+ Mosaics project [22] and Google Earth images obtained in 2012 or later. Those points that fell within salt pans, lakes, marshes, or other azonal land cover types, were manually relocated within a radius of ~3 km from the original position, or eliminated if the azonal cover dominated the landscape. After the selection and relocation processes, we maintained 6,186 points, 4,340 classified as natural and 1,846 as cultivated.

We accounted for the spatial climatic variability by calculating the PPT:PET based on the “Ten Minute Climatology database”, which averages 1961–1990 monthly data [23]. PET was calculated using the Penman-Monteith algorithm [24]. By using PPT:PET instead of PPT, we approach the conditions or water environment experienced by the vegetation. See the regional location of transects in Baldi and Jobbágy [21].

### Vegetation functioning

We characterized vegetation productivity exclusively based on the “Enhanced Vegetation Index” (EVI) time series, produced by the Terra “Moderate Resolution Imaging Spectroradiometer” (MODIS) instrument [25]. By applying a single recording protocol in time and space, this remote sensing variable has been extensively used to track different processes that depend on the light absorbed by vegetation canopy, regardless its type or ecophysiological condition [26–30]. EVI would outperform the earlier “Normalized Difference Vegetation Index” (NDVI) by minimizing the atmospheric noise, the saturation effects of high biomass areas, the canopy background signal, and the effects of absorption by non-photosynthetic components of the leaves [25,29,31].

Following Xiao et al. "Vegetation Photosynthesis Model" [32] and others [33,34], we considered EVI as equal to fAPAR (i.e. the fraction of photosynthetically active radiation (PAR) absorbed by the photosynthetic active vegetation in the canopy). Under the Monteith light use efficiency (LUE) model [35,36], EVI constitutes a first step to calculate GPP (GPP = LUE * fAPAR* PAR). However, in the context of our study, we elude GPP calculation in order to avoid introducing errors and biases due to (1) the lack of direct measurements of LUE at landscape scales (encompassing multiple plant functional types) [37], (2) the disparity of PAR values among regions (ranging from 2,600 Mj*m^-2^*y^-1^ in Mesquite or India & Pakistan to 3,800 Mj*m^-2^*y^-1^ in NE Australia or Zambezi-Kalahari, S1 Fig), and (3) the compensating mechanisms between PAR values, LUE and season length on annual GPP among regions and plant types [38]

### Data processing and analysis

For the 6,186 sampling points, we downloaded EVI data from 2000 to 2012 (coded as MOD13Q1; spatial and temporal resolutions of 250 m and 16 days) from the ORNL “MODIS Global Subsets: Data Subsetting and Visualization” tool (www.daac.ornl.gov). We only considered EVI values with the highest quality (flagged as category VI)–representing 79% of the entire data set–, eliminating the potential noise from clouds and aerosols. We used the software TIMESAT v.3.1 to reconstruct the EVI time series [39]. This tool smoothes series by means of model functions that capture one or two cycles of growth and decline per year. We selected an adaptative Savitzky-Golay model. From the reconstructed series, we calculated seven functional metrics depicting magnitude, seasonality, and inter-annual variability of vegetation productivity (Table 1) [10,40–42].

**Table 1:** EVI-based functional metrics.

	Metric	Description
1	Mean EVI	Mean EVI value. Calculated as the average of the 2000–2012 annual mean values (same for metrics #2 to #6 but changing the focus annual value).
2	Maximum EVI	Average of maximum EVI values.
3	Minimum EVI	Average of minimum EVI values.
4	Intra-annual EVI CV	Average of the coefficient of variation values.
5	Peakness	Ratio between 10,000 * maximum EVI and length of the growing period (metrics #2 and #6) representing kurtosis. The higher the value, the acuter the peak.
6	Length of the growing season	Length, in time (days), between the beginning to the end of the growing seasons. Beginning and end are recorded when the fitted EVI curve crosses the minimum + 0.25 * range value within a single year.
7	Inter-annual EVI CV	Inter-annual coefficient of variation of the 2000–2012 mean annual EVI values.

The seven metrics depict the magnitude (metrics 1 to 3), seasonality (4 to 6), and inter-annual variability (7) of the“Enhanced Vegetation Index” (EVI), a proxy variable of vegetation productivity [27,28]. Metrics were based on Paruelo et al. [41], Jobbágy et al. [10], Eklundh and Jönsson [42], and Baldi et al. [40].

To obtain a first graphic description of the productivity/water availability relationship, we represented seasonal dynamics of EVI for four PPT:PET equal intervals (0.2 to 0.4 up to 0.8 to 1.0). At each sampling point, we averaged the reconstructed EVI values of the 23 dates per year provided by MOD13Q1 for the temporal series of 13 years. We then explored the relationship by regressing functional metrics against PPT:PET by means of additive models (L_2_ smoothing splines) for quantile regression [43,44]. We selected the 0.5 quantile (hereafter τ_50_) in order to provide a description of the effect of *X* on the central tendency of *Y*, and 0.9 and 0.1 quantiles (τ_90_ and τ_10_, respectively) to describe the behavior of the *Y* variable when *X* is the dominant constraining variable [45], trying to avoid the effects of unmeasured factors (such as nutrient availability depressing productivity levels) or any type of sub-optimal use. We employed τ_90_ to represent the healthy or permissive state of unmeasured factors in variables like the magnitude of productivity, which are maximized by water availability. The opposite occurs for the variability of productivity, which is likely minimized by water availability and thus τ_10_ is the logical option. We selected for all regressions a smoothing term λ = 0.5, which empirically implied a good compromise between the goodness of fit and model simplicity.

**Fig 2:**
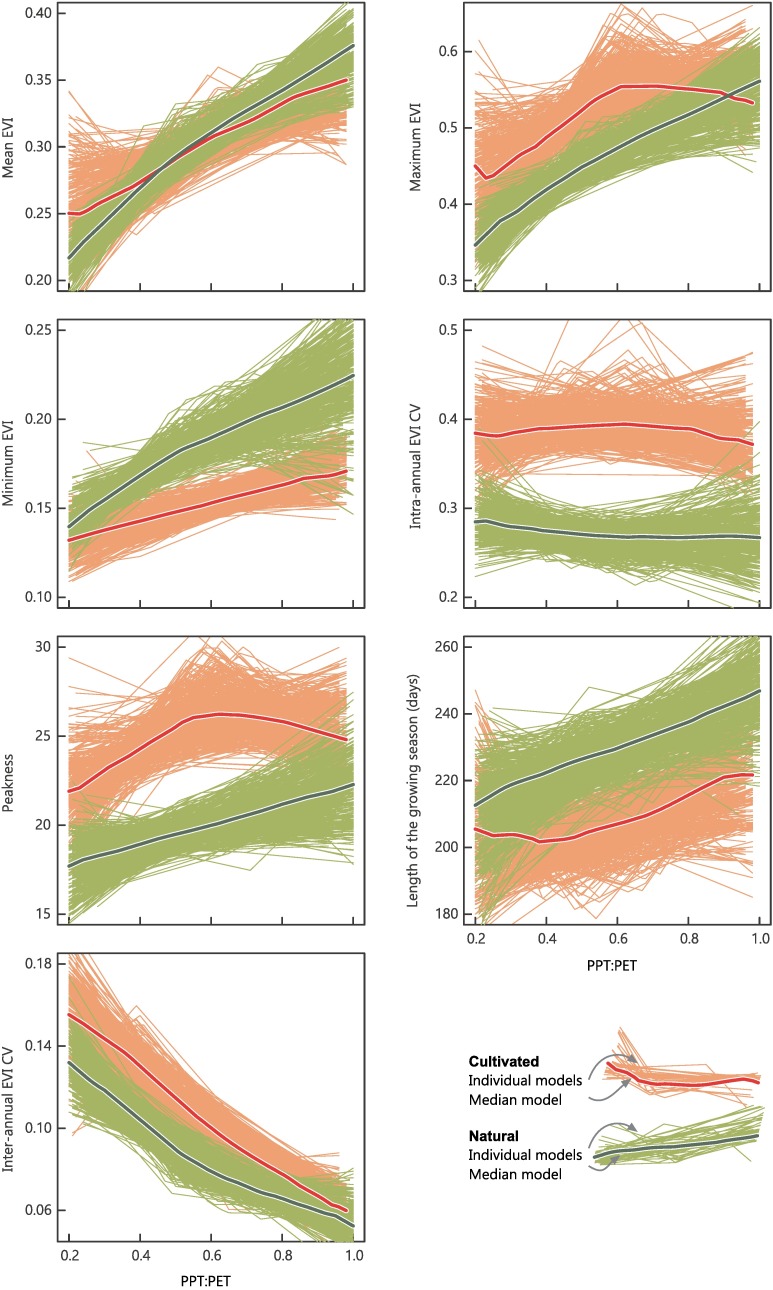
Median functional responses to water availability of natural vs. cultivated systems at the global level. Each panel represents the behavior of an EVI-based functional metric in relation to the PPT:PET. The thin lines represent the individual additive models for the 0.5 quantile (τ_50_) after a resampling approach (500 points). The thick line represents the averaging (with a median) of these individual models.

For the global level approach, we balanced sampling size differences among land use/cover systems and regions (S1 Table) by applying an *ad hoc* resampling method [46]. This implied that, for each system and region, (1) we randomly sampled five points within four equal intervals of the PPT:PET gradient (0.2 to 0.4 up to 0.8 to 1.0). (2) We fitted for each 100-points subsamples (5 points * 4 PPT:PET intervals * 5 regions) the additive models previously described and repeated this process 500 times. (3) We generated a median condition of all subsamples models (thick lines in Figs 2 and S3) by using the fitted data of individual models, and (4) we characterized the overall effects of cultivation by averaging along the PPT:PET gradient the fitted values (Table 2 and S2 Table). For the regional level approach, we repeated the additive model procedure but using the entire local set of points for each system, generating confidence bands (95%) along the PPT:PET gradient based on the Hotelling [47] tube approach. We used the fitted values from global and regional models to assess the net change on each functional metric. All processes were run in R (packages quantreg, MASS, splines, mgcv) (www.r-project.org). All information is available at the S1 Dataset.

**Table 2:** Median effect of cultivating the dry subtropics at global and regional levels.

Region	Mean EVI		Maximum EVI		Minimum EVI		Intra-annual EVI CV		Peakness		Length of the growing season		Inter-annual EVI CV	
	natural	cultivated	natural	cultivated	natural	Cultivated	natural	cultivated	natural	cultivated	natural	cultivated	natural	cultivated
Global	0.3 ± 0.04	0.3 ± 0.03	0.46 ± 0.06	0.52 ± 0.04	0.19 ± 0.02	0.15 ± 0.01	0.27 ± 0.01	0.39 ± 0.01	19.9 ± 1.3	24.9 ± 1.2	229 ± 8	208 ± 7	0.09 ± 0.02	0.11 ± 0.03
Chaco	0.36 ± 0.02	0.36 ± 0.02	0.48 ± 0.02	0.6 ± 0.06	0.23 ± 0.02	0.2 ± 0.03	0.22 ± 0.03	0.34 ± 0.08	18.6 ± 0.8	26.7 ± 5.5	257 ± 10	225 ± 23	0.08 ± 0.01	0.12 ± 0.02
India-Pakistan	0.29 ± 0.04	0.29 ± 0.03	0.55 ± 0.05	0.54 ± 0.04	0.15 ± 0.02	0.13 ± 0.01	0.44 ± 0.03	0.44 ± 0.02	31.3 ± 1.9	26.3 ± 1.2	176 ± 17	215 ± 7	0.08 ± 0.02	0.08 ± 0.01
Mesquite	0.29 ± 0.05	0.26 ± 0.02	0.4 ± 0.08	0.49 ± 0.04	0.18 ± 0.03	0.13 ± 0.01	0.23 ± 0.01	0.44 ± 0.1	15.1 ± 2.1	27.8 ± 9.1	264 ± 17	189 ± 7	0.13 ± 0.01	0.16 ± 0
NE Australia	0.24 ± 0.04	0.29 ± 0.14	0.35 ± 0.04	0.5 ± 0.15	0.17 ± 0.03	0.15 ± 0.1	0.22 ± 0.03	0.42 ± 0.14	17.4 ± 2.4	25.6 ± 8.5	201 ± 3	202 ± 36	0.11 ± 0.03	0.18 ± 0.07
Zambezi-Kalahari	0.3 ± 0.04	0.26 ± 0.02	0.46 ± 0.05	0.45 ± 0.05	0.17 ± 0.02	0.15 ± 0.01	0.3 ± 0.02	0.35 ± 0.03	20.4 ± 1.4	22 ± 2.2	224 ± 15	197 ± 2	0.07 ± 0.02	0.08 ± 0.02

Average and standard deviation of the seven EVI-based functional metricsof natural and cultivated systems, considering 0.5 quantile (τ_50_) additive models. In S2 Table, we show the summary information for the extreme effects of cultivating, considering τ_90_ and τ_10_ additive models. Acronym: CV, coefficient of variation.

## Results

**Fig 3:**
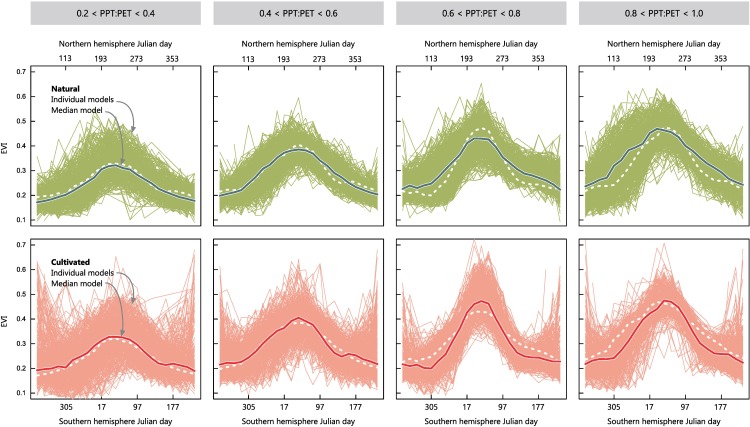
Median seasonal patterns of natural vs. cultivated systems at the global level. Each panel represents the seasonal behavior of an EVI-based functional metric within one of four equal PPT:PET intervals (0.2 to 0.4 up to 0.8 to 1.0) at the global level. The thin lines represent the individual additive models for the 0.5 quantile (τ_50_) after a resampling approach (500 points). The thick line represents the averaging (with a median) of these individual models. Upper panels represent natural systems, while lower ones, cultivated (dotted white lines symbolize the opposite system). Southern and Northern hemisphere sampling points were coordinated by shifting six months the data from one hemisphere.

When considering all regions together and the median distribution (τ_50_), natural and cultivated systems did not differ in terms of mean EVI (Table 2), displaying only slight variations along the PPT:PET gradient (cultivated surpassed natural systems towards drier conditions and vice versa towards more humid; crossover at PPT:PET = 0.53) (Fig 2). This notable convergence was achieved by cultivated systems through the increase of productivity peakness between PPT:PET 0.2 and 0.6, and through the extension of the growing season length at PPT:PET > 0.6 (Figs 2 and 3). Cultivation increased the annual maxima and decreased the annual minima of EVI (average τ_50_ +0.06 and-0.04, respectively). Initially, along the gradient of increasing water availability, maximum productivity grew in parallel on both systems; however, at PPT:PET > 0.6 a major functional change occurred with cultivation, since the maximum productivity of the implanted systems stabilized, diverging from the natural vegetation–which continued increasing up to the humid end of the gradient–(S2 Fig). The more extreme minimum EVI levels introduced by cultivation became more significant towards humid conditions, with models of cultivated systems showing the least pronounced slopes for this attribute along the water availability gradient (Fig 2). Regarding maximum EVI, we found that cultivation increased productivity peaks for theτ_90_ models, surpassing natural systems throughout the entire gradient (average differences: τ_90_ = +0.10 vs. τ_50_ = +0.06, Table 2 and S2 Table; S3 Fig).

As expected from the EVI extremes, the intra-annual CV for τ_50_ models was approximately one-third higher on cultivated systems compared to their natural counterparts (Table 2). Noticeably, intra-annual CV resulted more strongly affected by cultivation (average τ_50_ +0.12) than by water availability, showing little variation along the PPT:PET gradient (Fig 2). This behavior could be ascribed to a parallel increase of the seasonal variance–as suggested by the peakness–and the mean values (i.e. lower productivities coincide with wider seasonal curves) (Fig 3 and S3 Table). Only by exploring the change between models, a slight increase of variability was found around a PPT:PET = 0.6 (S2 Fig). Interestingly, the joint analysis of the intra-annual CV and seasonal dynamics (depicting an averaged behavior) (Figs 2 and 3), indicated that even absolute CV values remained invariant, the synchronization of individual curves of cultivated points acquired its maximum between a PPT:PET of 0.6 and 0.8. Cultivated systems showed the highest peakness (20% more acute shape) and a reduced growing season length (-21 days on average), resulting from longer lapses of low photosynthetic activity balanced by a delayed but accelerated and coordinated greening followed by an anticipated browning (Table 2 and Figs 2 and 3)–unlike natural systems, where both metrics increased linearly along PPT:PET gradients–. Cultivated systems also showed productivity peaks in winter/dry periods, unseen in natural systems.

Inter-annual EVI CV was a fifth higher on cultivated systems along the 13-year period (average CV_unc_ = 0.09 vs. CV_cul_ = 0.11, Table 2), being highly sensitive to water availability conditions (the drier the more unstable). Differences between systems were, however, shortened towards the humid end of the gradient, mainly due to the partial stabilization of natural systems above PPT:PET = 0.6 (S2 Fig). These patterns changed radically in the extreme τ_10_ models, with natural systems being more unstable than cultivated systems towards arid conditions, and all the way around towards the humid (S2 and S3 Figs).

Regional results generally sustained the global patterns in terms of average contrasts between systems (Table 2), yet loosely in the shape of the responses to water availability (S4 and S5 Figs). Notable departures arose for cultivated systems, especially in India & Pakistan and Zambezi-Kalahari. In the Asian region, cultivation led to higher mean values, wider curves, and longer growing seasons over most of the PPT:PET gradient. In the African region, the systems converged for the maximum and inter-annual CV EVI (τ_50_ and τ_90_ models) resulting from a relatively low productivity of cultivated systems. At last, contrary to the global behavior (τ_50_ and τ_10_ models), we found that the intra-annual CV markedly decreased towards humid conditions in Chaco and NE Australia.

## Discussion

Our study, encompassing a broad spatial and temporal range (6.4x10^6^ km^2^, five continents, 13 years of data), revealed that deforestation and subsequent agricultural expansion in the dry subtropics with summer rains appears to have a nil effect on the productivity of vegetation–given by the mean EVI–(Table 2), in line with previous remote sensing and eddy covariance assessments [7,48]. This contrasts with the generalized idea that this land use/cover change leads to a major functional degradation, with high risks of productive failure and misuse of resources in the short term [49]. Possibly, this outcome arises from the counterbalance of cultivated/natural vegetation advantages: the evolutionary adaptations of natural systems involving higher water use efficiency and stress tolerance vs. the human-selected adaptations of cultivated systems involving lower respiration costs and better responses to the environment of disturbances and subsidies (e.g. fertilizers, pesticides) [50]. Whichever the specific causes, we found that cultivated systems tracked natural ones in their steady increase of mean EVI along the entire water availability gradient (Fig 2). From dry to intermediate conditions, cultivation led to a progressive concentration of photosynthetic activity within a short growing season, and from intermediate to humid conditions, to an extension (or multiplication) of the growing season. This would reveal a mechanism through which humans, even when deeply intervening land ecosystems, tend to foster the exhaustive use of rainfall inputs.

The most prominent change brought by cultivation was a generalized increase in seasonality (captured by the intra-annual EVI CV), independently from the water availability conditions (Fig 2). This amplification was associated with an accentuation of EVI extremes and a shortening of the productive period–as shown by the length of the growing season and the peakness–(S3 Table). Unlike for natural vegetation, the latter functional traits maintained a non-linear relationship with water availability in cultivated systems, implying the existence of environments of a maximum functional impact after deforestation (especially at 0.5 to 0.6 of PPT:PET, S2 Fig). Notably, these conditions occurred in the more intensively cultivated areas within each region, e.g. the subhumid Chaco [21]. We relate these functional and land use/cover patterns to the dominance of pastures and the reliance on irrigation and fertilization of cropping activities towards the dry extreme of the gradient [49,51], and the increasing water-logging and biotic stresses for crops towards humid areas [52].

Cultivation also increased significantly the variability of production from year to year (Table 2) but less so towards more humid conditions (Fig 2), supporting previous assessments [11,12]. According to Paruelo and Lauenroth [53] and Volante et al. [7], this impact would be mostly related to the changes in the magnitude of the peak of productivity rather than to the changes in the growing season length. From a managerial perspective, we relate these results to the dependence of farmers decisions on fluctuating climatic and economic signals [53], especially in regions dominated by large-scale production systems oriented to regional/global markets (e.g. Chaco, Mesquite, and NE Australia, Table 2) [40]. On the contrary, smallholders would stabilize productivity through a diversified management that offers a more constant food supply (e.g. Zambezi-Kalahari). We acknowledge that the productive structure of landscapes could affect variability results, as stability might arise from the statistical averaging of several small-size paddocks within a MODIS pixel, potentially blurring the values obtained for India & Pakistan and Zambezi-Kalahari results [54].

From a morphological/ecophysiological perspective, the differences in inter-annual variability between systems could also be ascribed to human-selected vs. nature-selected plant adaptations. Native species display hydraulic redistribution, have larger rooting depths, slow-growth strategies, and particular structural tissues that confer individuals a high water use efficiency and stress tolerance, allowing for a stable production in dry and wet years [55,56]. Oppositely, cultivated species growth and production respond more rapidly to higher water availability in wet years, but experience higher risk of failure in dry years [57]. Beyond species-specific traits, cultivated systems have lower functional diversity, implying a larger vulnerability to disturbances and environmental fluctuations [15].

Undoubtedly, the conversion of natural vegetation increased the appropriation of vegetation productivity for human consumption [7,17]. Nevertheless, land use/cover transformation influences virtually all natural processes, jeopardizing the long-term provision of other ecosystem services [49]. Due to the dry climate and very flat topography of the encompassed regions, the arisen seasonal concentration of vegetation productivity and the lower mean productivity under humid conditions (Fig 2) may imply a partial consumption of incoming water, potentially triggering flooding and soil salinization processes [58]. Likewise, the significant lower minima values brought by cultivation, together with the higher inter-annual variability, represent an increased exposure of soils to erosion [59]. Beyond structural modifications, the accentuated temporal dynamics of productivity would affect the faunal composition and abundance of cultivated lands and surroundings by modifying the characteristics and the dynamics of habitat and resource availability [60].

Our global findings were influenced by unconsidered biophysical factors and by current and past management strategies and legacies on natural and cultivated vegetation [61]. By exploring extreme quantiles (τ_90_ and τ_10_), we revealed at the global level that cultivated systems can display the highest long-term stability under the driest conditions (S3 Fig). Regional results supported this for the extensively irrigated and fertilized India & Pakistan, but also for the rain-fed technified Mesquite and the non-technified Zambezi-Kalahari (Table 2 and S2 Table and S5 Fig) [62]. The condition or degradation status of the natural systems being replaced, together with the characteristics of the implanted cultivated systems, seem crucial determining the net effects of cultivation on productivity. In India & Pakistan and NE Australia, the combination of a degraded natural vegetation and a technified agriculture [63,64] would be responsible for the increase in mean EVI and the expansion of the growing season with cultivation. A high demand for forest products and livestock pressure [65], and the extensive aggressive mechanized clearance campaigns [66] are likely responsible for the low productivity of natural systems in these two regions.

Even though this paper refers to vegetation productivity, we recognize that translating remote sensing radiometric variables such as EVI into accurate gross primary productivity (GPP) or net primary productivity (NPP) remains a major challenge. Regarding annual GPP, uncertainties are related to the apparent need of site-specific empirical scaled EVI-fAPAR functions [67], and the variability of annual LUE (affected by vegetation structural and functional traits–e.g. photosynthetic syndrome–, and soil and climatic conditions) [67–72], sometimes solved by means of look-up tables (based on biome type and climatology) [37,73] or more recently by means of the carotenoid-sensitive “Photochemical Reflectance Index” (PRI) [38,74,75]. However, the accuracy of annual EVI-GPP relationships seems to improve in land covers with high annual EVI ranges and summer rainfalls [28], as in our case. Regarding NPP, even larger uncertainties emerge, particularly those coming from the disparate respiration rates of foliage, stem, and roots [76,77], with a still elusive quantification over large extensions and contrasting plant functional types. In the dry subtropics, despite limited direct measures of GPP or NPP from long-term controlled field experiments (e.g. biomass harvests or flux towers), evidence shows that cultivated systems achieved the highest maximum daily rates of photosynthetic uptakes, but this difference is compensated by the temporality of production [17,48].

## Conclusion

Our remote sensing approach provides a new quantitative insight on the relative productive differences between original natural woody systems and novel cultivated systems in dry summer-rains subtropics. Cultivation increased the seasonality and the inter-annual variability of vegetation productivity without affecting its magnitude, which responded mainly to water availability. Climatic water availability is important determining productivity in natural systems; however, it loses its strength in cultivated systems, which seem to saturate their productivity at dry subhumid conditions (PPT:PET around 0.5). In the last decades, many researchers have explored the physical constraints of vegetation productivity in order to predict its response to global climate change. Using similar data and methodologies, we assess a different dimension of change, driving the functional debate towards the effects of the ubiquitous and accelerated land use/cover shifts. We highlight the considerable changes in seasonal vegetation activity and long-term variability (with likely parallels on carbon, water, and surface energy exchange) and reveal that the implications of the land transformations depend both on the physical and human contexts (accounted here by the water availability gradients and by the regions).

## Supporting Information

S1 Dataset In Baldi_et al_Sup Inf (data).xlsx, all EVI values and EVI metrics data are available. (XLSX)

S1 Fig Regional differences in the precipitation and the photosynthetically active radiation (PAR). We calculated precipitation and PAR (MJ*m^-2^) from the Climatic Research Unit–UEA “Ten Minute Climatology” data base [23], with a spatial resolution of 10 min (1961–1990 period). For PAR, average values about the sunshine (fraction of maximum daylength) were transformed using the Allen et al. [24] algorithms, and considering the ratio between radiation and PAR as 0.48 according to Tsubo and Walker [78] for a dry subtropical, summer-rain climate.
(EPS)

S2 Fig The absolute effect of cultivating the dry subtropics at the global level. Each panel represents the difference between cultivated and natural additive models for the 0.5 and 0.9 or 0.1 quantiles (τ_50_ and τ_90_ or τ_10_) in relation to PPT:PET. Numbers within panels indicate average values about the differences between land use/cover systems according to the τ_50_ and τ_90_or τ_10_ models. Data came from the global additive median models (fitted values) for the seven EVI-based functional metrics.
(EPS)

S3 Fig Extreme functional responses to water availability of natural vs. cultivated systems at the global level. Each panel represents the behavior of an EVI-based functional metric in relation to the PPT:PET. The thin lines represent the individual additive models for the 0.9 or 0.1 quantiles (τ_90_ or τ_10_) after a resampling approach (500 points). The thick line represents the averaging (with a median) of these individual models.
(EPS)

S4 Fig Median functional responses to water availability of natural vs. cultivated systems at the regional level. Each panel represents the behavior of an EVI-based functional metric and region in relation to the PPT:PET. Each dot represents a sampling point and each line an additive 0.5 quantile (τ_50_) model. Note that not all regions cover the entire water availability gradient. Gray bands indicate 95% confidence intervals according to the Hotelling [47] tube approach.
(EPS)

S5 Fig Extreme functional responses to water availability of natural vs. cultivated systems at the regional level. Each panel represents the behavior of an EVI-based functional metric and region in relation to the PPT:PET. Each dot represents a sampling point and each line an additive 0.9 or 0.1 quantiles (τ_90_ or τ_10_) model. Note that not all regions cover the entire water availability gradient. Gray bands indicate 95% confidence intervals according to the Hotelling [47] tube approach.
(EPS)

S1 Table Sampling details (transects and points) across the dry subtropics receiving summer rains. (DOC)

S2 Table Extreme effects of cultivating the dry subtropics at global and regional levels. Average and standard deviation values for the seven EVI-based functional metrics showing 0.9quantile (τ_90_) and 0.9quantile (τ_10_) additive median models of natural and cultivated systems (fitted values in S3 Fig). Acronym: CV, coefficient of variation.
(DOC)

S3 Table Kendall’s τ non-parametric correlation coefficients among functional metrics. (DOC)
