# Supplementary material for: Vegetation Productivity in Natural vs. Cultivated Systems along Water Availability Gradients in the Dry Subtropics
Source: PLoS One. 2016 Dec 22;11(12):e0168168. doi: 10.1371/journal.pone.0168168 (PMC5179098; doi:10.1371/journal.pone.0168168)
Supplement: S1 Table — (DOC) [file pone.0168168.s007.doc]

| **Region** | **Transects** | **Cultivated points** | **Natural points** |
| --- | --- | --- | --- |
| Chaco | 8 | 414 | 1134 |
| India & Pakistan | 7 | 802 | 172 |
| Mesquite | 3 | 97 | 203 |
| NE Australia | 6 | 90 | 871 |
| Zambezi-Kalahari | 11 | 443 | 1960 |
| Total | 35 | 1846 | 4340 |
