# Supplementary material for: Vegetation Productivity in Natural vs. Cultivated Systems along Water Availability Gradients in the Dry Subtropics
Source: PLoS One. 2016 Dec 22;11(12):e0168168. doi: 10.1371/journal.pone.0168168 (PMC5179098; doi:10.1371/journal.pone.0168168)
Supplement: S2 Table — Average and standard deviation values for the seven EVI-based functional metrics showing 0.9quantile (τ90) and 0.9quantile (τ10) additive median models of natural and cultivated systems (fitted values in S3 Fig). Acronym: CV, coefficient of variation. (DOC) [file pone.0168168.s008.doc]

| **Region** | **Mean EVI (**τ_90_) | | **Maximum EVI (**τ_90_) | | **Minimum EVI (**τ_90_) | | **Intra-annual EVI CV**  **(**τ_10_) | | **Peakness (**τ_90_) | | **Length of the growing season (**τ_90_) | | **Inter-annual EVI CV (**τ_10_) | |
| --- | --- | --- | --- | --- | --- | --- | --- | --- | --- | --- | --- | --- | --- | --- |
|  | **natural** | **cultivated** | **natural** | **cultivated** | **natural** | **cultivated** | **natural** | **cultivated** | **natural** | **cultivated** | **natural** | **cultivated** | **natural** | **cultivated** |
| Global | 0.38 ± 0.03 | 0.38 ± 0.02 | 0.59 ± 0.05 | 0.69 ± 0.04 | 0.26 ± 0.04 | 0.23 ± 0.03 | 0.17 ± 0.01 | 0.25 ± 0.02 | 32.3 ± 0.9 | 38.3 ± 3.9 | 272 ± 5 | 254 ± 8 | 0.05 ± 0.01 | 0.06 ± 0.01 |
| Chaco | 0.42 ± 0.04 | 0.42 ± 0.04 | 0.59 ± 0.05 | 0.71 ± 0.08 | 0.29 ± 0.04 | 0.26 ± 0.04 | 0.17 ± 0.03 | 0.23 ± 0.04 | 23.4 ± 1.2 | 38.0 ± 9.1 | 276 ± 12 | 263 ± 11 | 0.05 ± 0.01 | 0.07 ± 0.02 |
| India-Pakistan | 0.34 ± 0.02 | 0.37 ± 0.01 | 0.66 ± 0.01 | 0.69 ± 0.02 | 0.19 ± 0.03 | 0.19 ± 0.01 | 0.32 ± 0.00 | 0.31 ± 0.00 | 40.3 ± 2.9 | 35.8 ± 4.3 | 205 ± 17 | 244 ± 2 | 0.06 ± 0.01 | 0.05 ± 0.00 |
| Mesquite | 0.33 ± 0.06 | 0.32 ± 0.02 | 0.46 ± 0.06 | 0.6 ± 0.01 | 0.22 ± 0.04 | 0.16 ± 0.00 | 0.18 ± 0.00 | 0.31 ± 0.03 | 18.0 ± 2.1 | 39.1 ± 9.1 | 285 ± 1 | 245 ± 10 | 0.10 ± 0.01 | 0.11 ± 0.02 |
| NE Australia | 0.28 ± 0.05 | 0.43 ± 0.14 | 0.43 ± 0.04 | 0.71 ± 0.04 | 0.23 ± 0.04 | 0.22 ± 0.13 | 0.31 ± 0.03 | 0.12 ± 0.01 | 23.7 ± 2.5 | 32.3 ± 3.0 | 250 ± 8 | 242 ± 43 | 0.06 ± 0.01 | 0.1 ± 0.05 |
| Zambezi-Kalahari | 0.36 ± 0.05 | 0.32 ± 0.02 | 0.56 ± 0.07 | 0.57 ± 0.06 | 0.23 ± 0.04 | 0.18 ± 0.00 | 0.23 ± 0.03 | 0.26 ± 0.03 | 26.4 ± 2.9 | 29.9 ± 3.3 | 251 ± 16 | 229 ± 7 | 0.05 ± 0.02 | 0.05 ± 0.01 |
