# Supplementary material for: Vegetation Productivity in Natural vs. Cultivated Systems along Water Availability Gradients in the Dry Subtropics
Source: PLoS One. 2016 Dec 22;11(12):e0168168. doi: 10.1371/journal.pone.0168168 (PMC5179098; doi:10.1371/journal.pone.0168168)
Supplement: S3 Table — (DOC) [file pone.0168168.s009.doc]

|  | Mean EVI | Maximum EVI | Minimum EVI | Intra-annual EVI CV | Peakness | Length of the growing season |
| --- | --- | --- | --- | --- | --- | --- |
| Maximum EVI | 0.62 |  |  |  |  |  |
| Minimum EVI | 0.54 | 0.19 |  |  |  |  |
| Intra-annual EVI CV | -0.06 | 0.32 | -0.48 |  |  |  |
| Peakness | 0.27 | 0.63 | -0.08 | 0.56 |  |  |
| Length of the growing season | 0.37 | 0.04 | 0.44 | -0.44 | -0.33 |  |
| Inter-annual EVI CV | -0.25 | -0.12 | -0.24 | 0.15 | 0.03 | -0.24 |
